# Vision drives the neural construction of a two-stage hierarchy of spatial processing in infancy

**DOI:** 10.1016/j.isci.2025.113707

**Published:** 2025-10-04

**Authors:** Monica Gori, Helene Vitali, Andrew J. Bremner, Alessia Tonelli, Maria Bianca Amadeo, Walter Setti, Carolina Tammurello, Sabrina Signorini, Elena Cocchi, Giuseppina Giammari, Sandra Strazzer, Francesca Tinelli, Massimiliano Serafino, Paola Camicione, Claudio Campus

**Affiliations:** 1 Unit for Visually Impaired People (U-VIP), Istituto Italiano di Tecnologia, 16152 Genoa, Italy; 2 DIBRIS, University of Genova, Genoa, Italy; 3 Centre for Developmental Science, School of Psychology, University of Birmingham, Birmingham, UK; 4 Department of Psychology, The University of Sydney, Sydney, NSW, Australia; 5 Developmental Neurophthalmology Unit, IRCCS Fondazione Mondino, Pavia, Italy; 6 Fondazione David Chiossone, Genoa, Italy; 7 Scientific Institute, IRCCS E. Medea, Bosisio Parini, Lecco, Italy; 8 Department of Developmental Neuroscience, IRCCS Fondazione Stella Maris, Pisa, Italy; 9 IRCSS Istituto Giannina Gaslini, Genoa, Italy

**Keywords:** Neuroscience, Developmental neuroscience, Sensory neuroscience

## Abstract

Here, we demonstrate the critical role of developmental vision in the origins of a neural processing hierarchy in which somatosensory events are mapped from somatotopic locations onto a body representation with respect to external space. Both sighted and severely visually impaired infants showed prominent contralateral somatosensory activation in uncrossed- and crossed-hands postures in the early feedforward stages of processing (45–65 ms). By 105–120 ms following the somatosensory stimulus, anatomically ipsilateral activation was observed in the crossed-hands posture in sighted infants only, which was directly related to behavioral orienting toward the incorrect hand, reflecting a remapping of touches with reference to the position of the limbs in external space. Severely visually impaired infants exhibited a somatosensory response that was persistent contralateral across early and later processing stages, with the early responses associated with behavior. These findings demonstrate how visual experience in early postnatal development constructs the neural-behavioral basis of embodied spatial perception.

## Highlights

• Vision drives the remapping of touch from body-based to external space in infancy
• Blind infants rely on anatomical coding during early somatosensory processing
• Sighted infants show posture-sensitive remapping in later somatosensory stages
• Auditory responses remain spatially stable across posture in both groups

## Graphical abstract

**Figure:**
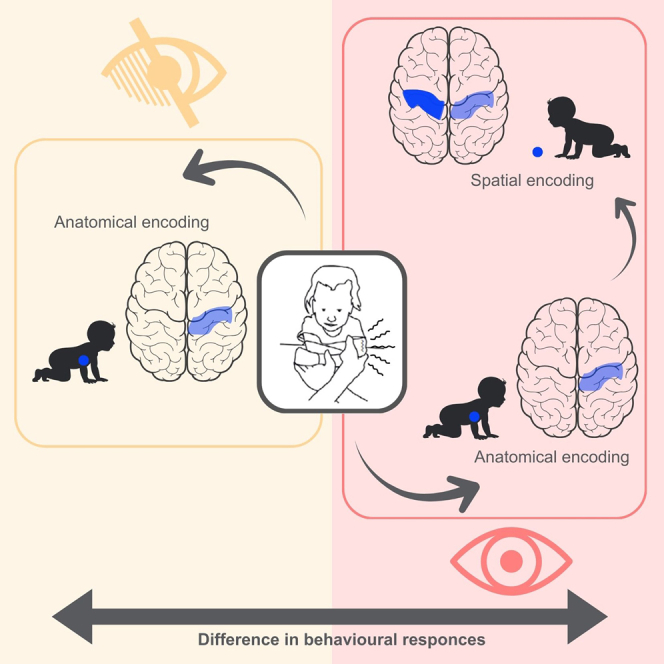

## Introduction

The way sighted human infants respond to spatial somatosensory events undergoes a fundamental developmental reorganisation in the first postnatal year. Up until 4 months of age, infants locate touches on the limbs according to anatomical coordinates, and only in the latter part of the first year do they represent touches with reference to external spatial coordinates, taking account of limb position across dynamic changes in posture.^1^^,^^2^^,^^3^

This developmental pattern in which tactile spatial sensing changes from anatomical coding to external referential coding (either with respect to external spatial coordinates or the canonical/default position of the limbs with respect to external space) is echoed by a two-stage spatial processing hierarchy seen in adults.^4^^,^^5^^,^^6^ When locating a touch, adults first prioritize somatotopic (anatomical) coordinates, but within 200 ms reprioritize spatial coordinates which locate the touch with respect to external space (We note that there is a vibrant discussion concerning whether human somatosensory maps touches to locations in external space or to locations on the body with reference to the canonical layout of the limbs in external space. In this article, we do not distinguish between these accounts, but note that both of these accounts require the registration of touches with reference to representations of an external frame of reference, which goes beyond somatotopy or bodily anatomy^5^^,^^7^.),^2^^,^^4^^,^^5^^,^^6^^,^^8^^,^^9^^,^^10^^,^^7^^,^^11^ spatial representational formats which better support skilled action in three-dimensional multisensory space.^12^

Indeed, when adults attempt to locate a tactile stimulus on the hand during a crossed-hands posture, in some situations, the touch is not attributed to the stimulated hand but rather to the hand resting where the touch would normally occur on the opposite side of external space.^4^^,^^5^ These remapping phenomena are evident both in behavior and neural responses. Specifically, the process of localizing a touch on the body entails a double mechanism. First, the stimulus is localized on the body, and later, it is mapped onto external space,^13^ a spatial representational format that better supports skilled action in three-dimensional multisensory space.^12^ Brain-imaging techniques demonstrate the somatosensory neural processing hierarchy underlying this two-step process, with an early period, around ∼50 ms, during which somatotopic activation reflects anatomical coordinates^8^^,^^9^^,^^14^; and a subsequent later period, around ∼100 ms, that marks the completion of a shift to a somatotopy reflecting the position of somatosensory stimuli on the limbs in external spatial coordinates.^14^^,^^15^^,^^16^^,^^17^ The first step reflects the activity of the primary somatosensory cortex (SI).^18^^,^^19^^,^^20^ Then, activity extends to the secondary somatosensory cortex (SII), posterior parietal regions (PPC), and frontal areas.^21^ The involvement of SII and its associated network is likely the phase during which remapping with respect to limb position in external space occurs, probably mediated by back-projections from the PPC to somatosensory cortices.^22^^,^^23^^,^^24^

The development of this somatosensory processing hierarchy, as well as the ability to remap somatosensory locations across changes in posture, seems to be influenced by visual experience. Indeed, at 10 months of age, visual cues from one’s own body allow the child to account for body position.^2^^,^^11^ Moreover, research with congenital, early, and late blind adults has shown that early visual experience plays a critical role in developing differences in multisensory spatial processing profiles, and in behavioral markers of the remapping of touch with respect to external spatial coordinates, e.g., the crossed-hand effect, which in fact is absent in congenital blind individuals.^25^^,^^26^^,^^27^ Therefore, investigating spatial processing in visually impaired infants offers excellent promise for tracing the role of sensory experience in the early development of spatial sensing in the brain and behavior.

Despite the difficulties involved in experimental work with severely visually impaired infants (fewer than 3 in 10,000 live births), a recent study has shown that, in contrast to sighted infants, congenitally blind infants show no influence of external space on their behavioral orienting to touches,^2^ even leading them to outperform sighted infants when orienting to a touch when their hands are crossed over (the sighted infants made mistakes in the crossed-hands posture, likely due to their remapping of touches on their limbs with respect to external coordinates). However, there are considerable challenges in interpreting behavioral findings from human infants because we do not know whether behavioral developments are occurring at sensory, cognitive, or motor stages of processing. For instance, it is possible that differences between sighted and SVI infants in Gori et al.’s study were due to differences in either spatial sensing or spatial orienting. In this study, we use EEG in order to open a window onto the development of the sensory brain. We focus specifically on uncovering whether differences in spatial representation between blind and sighted infants rest on differences in the early feedforward stages of sensory processing.

We used a combination of electroencephalography (EEG) and behavioral measures with sighted (S) and severely visually impaired (SVI) infants to investigate a neural spatial anatomical-to-external processing hierarchy subserving spatial processing seen in adults,^9^^,^^14^^,^^15^^,^^16^^,^^17^^,^^28^ and the role of visual experience in its developmental construction in early life. We hypothesized that neural processes related to somatosensory perception and body remapping are modulated by vision with a specific functional temporal hierarchy. Therefore, we expected to observe a well-developed two-stage anatomical-to-external spatial processing hierarchy in the S group, while altered neural patterns may be observed in the SVI group due to the absence of visual experience. Conversely, we predicted that limb position may not noticeably impact brain responses to auditory stimulation, considering that auditory stimuli are directly mapped on the external reference frame from the first stages of sensory processing in both groups of infants. Our results reveal the early developmental origins of a neural processing hierarchy that underpins the mapping of cutaneous tactile events from anatomical to external spatial reference frames and show that its construction depends on visual experience.

## Results

We tested a group of SVI infants (*n* = 12, 6 females, median age = 28.1 months, age range = 10–54 months) and a group of age-matched sighted (S) infants (*n* = 12, 5 females, median age = 31 months, age range = 12–52 months). Participant demographic and clinical details are reported in Table S1. We administered each group with a set of trials in which a single stimulus (auditory or tactile) was presented on one of their hands. On each trial, the participant’s hands were either crossed or uncrossed. We then recorded event-related potentials (ERPs) gathered from scalp EEG, and behavioral orienting responses were recorded and coded from video records. We defined responses as contralateral or ipsilateral relative to the stimulated hand, regardless of its spatial position. As expected, somatosensory responses were predominantly contralateral to the stimulated hand, while auditory responses were more contralateral to the side (i.e., spatial position) of the stimulated hand. When hands were uncrossed, the hand and its side position aligned (e.g., the left hand on the left side). In this case, a contralateral response occurred in the right hemisphere, consistent with both the hand and its side. However, in the crossed hands condition, the side and the stimulated hand were dissociated (e.g., the left hand positioned on the right side). For auditory stimuli in this configuration, we observed responses that were contralateral to the side (i.e., in the left hemisphere), which was therefore ipsilateral to the stimulated hand. For example, when stimulating the left hand, we defined the right hemisphere as contralateral, regardless of hand position. But when that hand was placed on the right side (crossed posture), the resulting ERP was more contralateral to the physical location of the sound (i.e., in the left hemisphere), which is ipsilateral to the stimulated hand. The main results are shown in Figures 2, 3, and 4.

### Behavioral orienting responses

**Figure 1:**
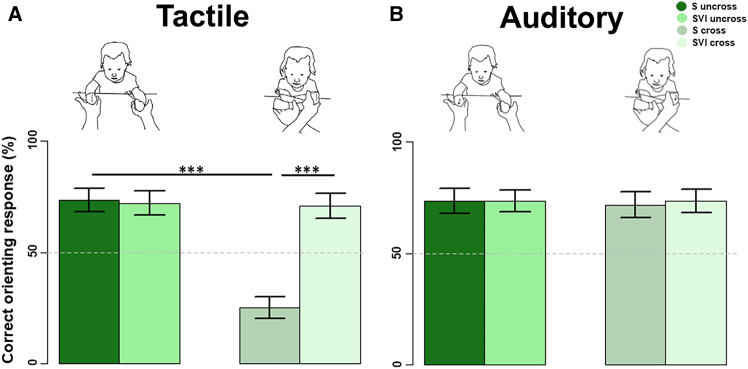
Percentage accuracy of S and SVI infants’ orienting responses in the tactile and auditory stimulus conditions and across posture conditions The bars represent the percentages of head, eye, and manual orienting responses made toward the hand that received the stimulus (mean and sem). The asterisks represent significant *p*-values after Bonferroni correction (∗ = *p* < 0.05, ∗∗ = *p* < 0.01, ∗∗∗ = *p* < 0.001). (A) Tactile only condition. (B) Auditory only condition.

A response was considered correct when its direction was toward (congruent with) the stimulated hand, irrespective of stimulus location in space). We applied generalised linear mixed models (GLMMs) evaluated using Wald χ2. Significant fixed effects were further investigated with contrasts based on t tests. The behavioral results (see Figure 1 and Supplementary Materials Tables S2 and S3) confirmed previous findings with S and SVI infants.^2^ There was no reliable effect of Group (S/SVI) or Posture (Uncrossed-hands/Crossed-hands) on the accuracy of behavioral orienting responses to auditory stimuli. However, in response to tactile stimuli, whereas the S infants showed poorer accuracy (*p* < 0.001) when their hands were crossed than when they were uncrossed, the SVI infants performed comparably with the best performance of the S infants in both uncrossed- and crossed-hands postures (see Figure 1; Table S3).

### Somatosensory event-related potentials

**Figure 2:**
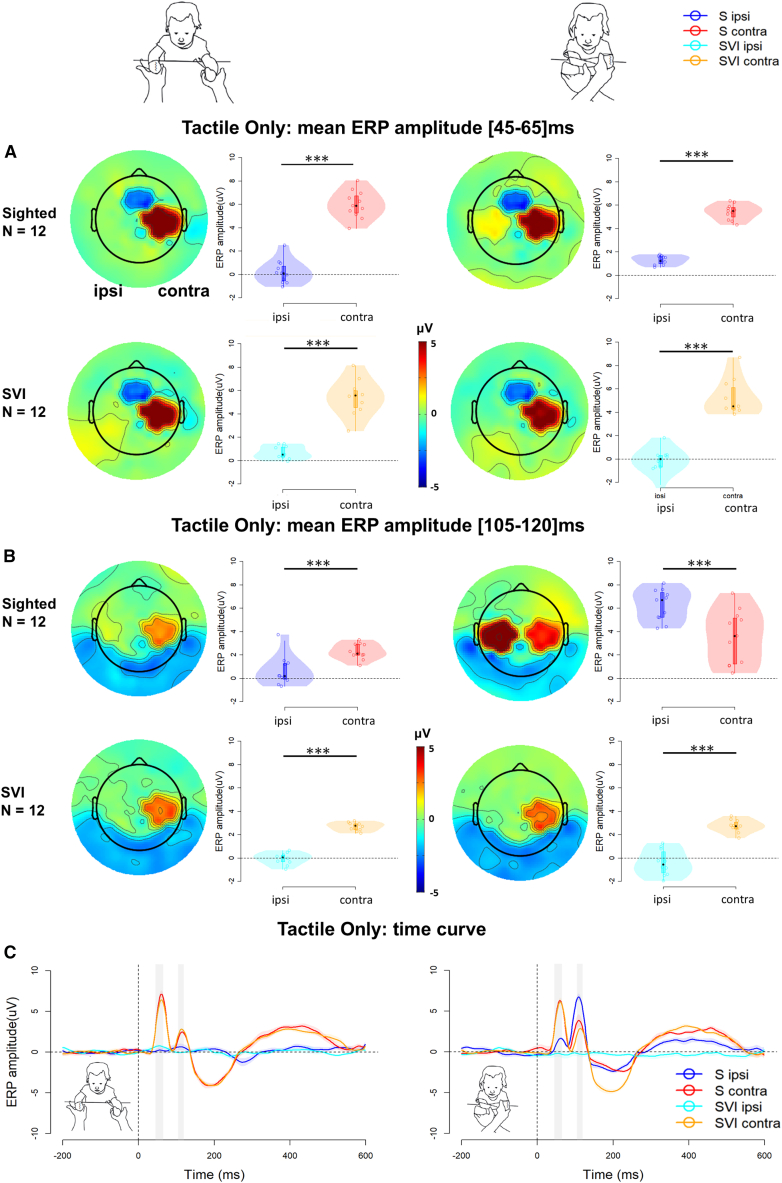
ERPs in the tactile only condition across posture conditions and groups (S/SVI) (A and B) represent amplitude differences in the [45–65]ms and [105–120]ms time windows, respectively. For each Group (row) and Posture (column) we report, on the left, the topography distribution, and, on the right, the mean ERP amplitude (violin plot), the median (boxplot), and related 95% CI (vertical line), and the single-subjects ERP amplitude (scattered plot). The horizontal dashed line points out 0 amplitude. The stars represent the significant *p* values (∗ = *p* < 0.05, ∗∗ = *p* < 0.01, ∗∗∗ = *p* < 0.001) after a Bonferroni correction for multiple comparisons. (C) represents the ERP curve for different groups and hemispheres during uncrossed (left panel) and crossed (right panel) postures. The horizontal and vertical dashed lines point out 0 amplitude and t = 0, respectively. The shadowed areas highlight the time windows where amplitudes were compared.

For each time window and posture, we evaluated differences in ERP amplitude according to group (SVI vs. S) and laterality (i.e., Ipsilateral vs. Contralateral hemisphere with respect to the stimulated hand). To this aim, we applied linear mixed models (LMMs) evaluated using Wald χ2. Significant fixed effects were further investigated with contrasts based on t tests (for detailed results, see Table S4). The somatosensory ERPs in both the SVI and S groups revealed a notable central-parietal positivity contralateral to the stimulated hand during two critical time windows: [45–65]ms and [105–120]ms, see Figures 2A and 2B, and Table S4. However, the temporal hierarchy of somatosensory processing shows a clear differentiation between S and SVI groups, particularly in the crossed-hands posture. In the early time window, both groups exhibited centroparietal positivity contralateral to the stimulated hand in the crossed-hands posture (Figures 2A and S1). The S infants already showed a weak ipsilateral activation as early as the [45–65]ms time window. However, in the [105–120]ms time window, evidence of somatosensory remapping is fully apparent in the S group only. In S infants, ipsilateral central-parietal positivity surpasses the contralateral response, whereas in the SVI infants, the response remains firmly contralateral across both postures. A cluster analysis of the infants’ EEG data, identifying microstates (i.e., quasi-stationary and functionally homogeneous states of the brain, see STAR Methods section) across auditory and somatosensory ERPs (see Figure S1), confirmed the functional significance of the investigated time windows and areas. This analysis reaffirmed that, in the crossed-hand condition, only sighted infants exhibited a remapping process of tactile information. This is evidenced by the early localization of the stimulus on the body, marked by a central activation contralateral to the stimulated hand, before being mapped onto external space with a transition to bilateral activation.

### Somatosensory behavioral association

**Figure 3:**
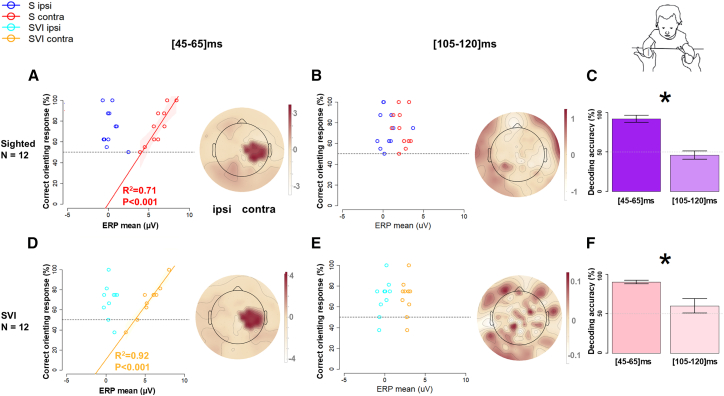
Tactile only condition, uncrossed hands posture: association between orienting response and EEG For each Group (row) and time window (column), (A), (B), (D), and (E) report the results of linear regression of orienting accuracy to ERP amplitudes (left) and MVPA classifying multivariate EEG against the accuracy of the response (right). The maps represent the feature of weight distribution over the scalp during the selected time window, with red and white colors respectively indicating positive and negative associations with orienting performance. The rightmost subplots, (C) and (F), represent the decoding performance of the classifier (mean and SD) within the time windows. Stars indicate a significant t-test between time windows (*p* < 0.05 after Bonferroni correction).

To investigate associations between the participants’ behavior and their ERP amplitudes, for each time window and posture, we applied Linear regression (LM) of ERP amplitude to a participant’s accuracy (%) across trials and Generalized Linear Mixed Effects Models (GLMMs) to trial response correctness (assuming correctness for responses congruent with the stimulated hand). GLMMs were evaluated using Wald χ2. Significant fixed effects were further investigated with contrasts based on Z tests (for detailed results, see Table S5). In both S and SVI children during the uncrossed hand posture, we identified a positive correlation between the mean ERP of contralateral central-parietal response and accuracy during the early time window (45-65 ms), respectively R^2^ = 0.71, *p* < 0.001 in S infants, and R^2^ = 0.92, *p* < 0.001 in SVI infants (see Figures 3A and 3D) with no group differences.

**Figure 4:**
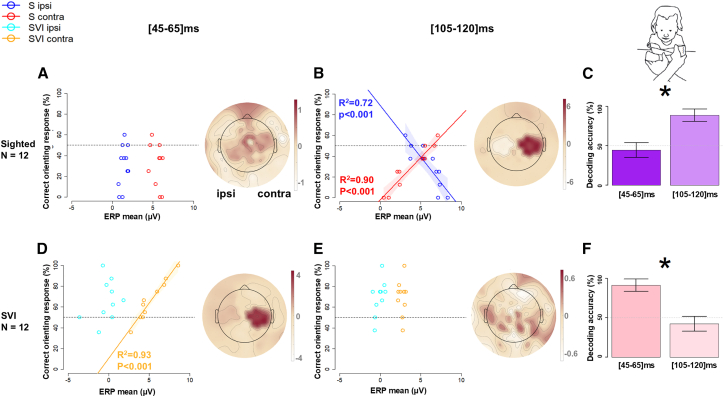
Tactile only condition, crossed hands posture: association between orienting response and EEG For each Group (row) and time window (column), (A), (B), (D), and (E) report the results of linear regression of orienting accuracy to ERP amplitudes (left) and MVPA classifying multivariate EEG against the accuracy of the response (right). The maps represent the feature of weight distribution over the scalp during the selected time window, with red and white colors respectively indicating positive and negative associations with orienting performance. The rightmost subplots, (C) and (E), represent the decoding performance of the classifier (mean and SD) within the time windows. Stars indicate a significant t-test between time windows (*p* < 0.05 after Bonferroni correction).

When in the crossed-hands posture, a significant positive correlation between mean ERP amplitude and orienting accuracy is observed in SVI children only in the early 45-65 ms (and not the late) time window (Figure 4D), R^2^ = 0.93, *p* < 0.001 (see also Table S4). For S infants, the mean amplitudes of contralateral and ipsilateral responses correlate with mean orienting accuracy (positive correlations between contralateral activation and accuracy, negative correlations between ipsilateral activations and accuracy) in the later 105-120 ms time window (Figure 4B), indicating that the ipsilateral remapping to external reference leads to decreases in the accuracy of behavioral orienting responses.

The close association of the amplitude of somatosensory ERP components with behavioral orienting performance was confirmed by GLMMs fitted for each time window (see Table S5), as well as by multivariate pattern analysis (MVPA) conducted on ERPs (see Figure 3). MVPA uses machine learning classification techniques to differentiate patterns of activation across a cluster of channels, e.g., in response to different stimuli.^29^

### Auditory event-related potentials

We observed a temporal-posterior positivity in response to auditory stimulation in the 45–65 ms time window, contralateral to the physical position of the sound (see Figures S2 and S3; Table S6). A positive correlation between ERP in the temporal area and behavioral accuracy was observed both in SVI and S infants (Figure S6; Table S6). Importantly, the absence of Group (SVI/S) and Posture (Crossed/Uncrossed) effects or interactions indicates that the auditory localization abilities of SVI infants were comparable to those of S infants, both in terms of behavior and neural processing (see Table S6).

## Discussion

This study underscores how vision shapes the development of tactile brain processes during early childhood. In both uncrossed and crossed-hand postures, an early somatosensory ERP response is observed in the central area anatomically contralateral to the stimulated hand. In this early defined component, 45–65 ms following the presentation of the somatosensory stimulus, SVI infants show a correlation between this early contralateral response and their behavioral orienting responses across both uncrossed- and crossed-hand postures, indicating a direct role of feedforward anatomical spatial coding on behavioral orienting. By 105–120 ms following the stimulus, the blind infants show no association between ERP amplitude and behavioral accuracy. Thus, in the case of blindness, infants' behavioral orienting to tactile stimuli is driven by an anatomical spatial code in the early feedforward stages of somatosensory processing. Sighted infants show a very different relationship between somatosensory processing and behavior, particularly in the crossed-hands posture. At the early feedforward stage of processing (45–65 ms), they only show the direct relationship between contralateral SEP amplitude and behavioral orienting accuracy in the uncrossed hands posture. At 120–205 ms, they show stronger activation in the hemisphere which is ipsilateral anatomically to the hand and contralateral in external space to their current hand position. This indicates that, following the earliest feedforward stage, the sighted infants were using a somatosensory spatial code that goes beyond anatomical coordinates, taking account of the position of their hand in external space.

**Figure 5:**
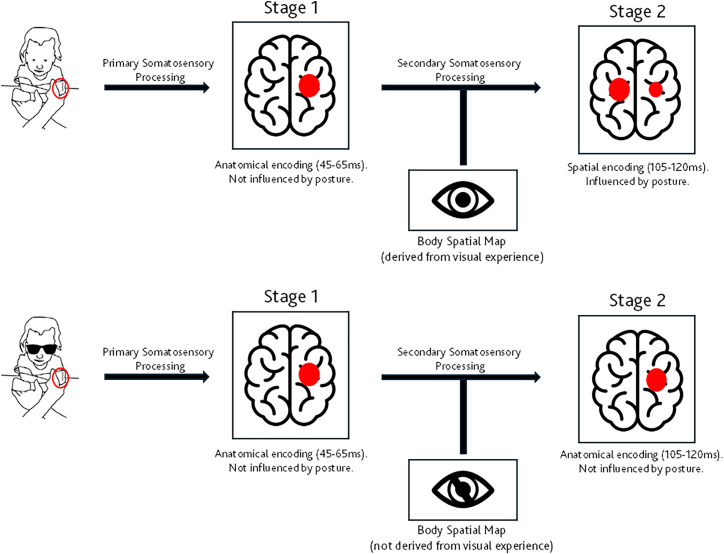
Theoretical model of the two-stage tactile processing framework Theoretical model of the two-stage tactile processing framework. On the top theoretical model of sighted infants. A tactile stimulus is first processed via Stage 1: Anatomical Encoding (45–65 ms), involving contralateral activation in the primary somatosensory cortex. This stage encodes the location of the touch on the body and is not influenced by posture. Subsequently, the signal proceeds to Stage 2: External Spatial Reference (105–120 ms), where bilateral activation reflects the remapping of tactile input into an external spatial reference frame. This second stage is modulated by visual experience, which provides contextual input via a feedback mechanism from a body spatial map derived from vision. The model highlights the integration of somatosensory and visual-derived spatial information during tactile perception. On the bottom theoretical model in SVI infants. No differences were evidenced by primary and secondary somatosensory processing due to the absence of visual experience.

Overall, these findings highlight the development of a two-stage anatomical to external spatial somatosensory processing hierarchy in sighted infants, as schematized in Figure 5. Blind infants, in contrast, appear to rely purely on anatomical spatial coding supported by early feedforward somatosensory processing. Critically, these data show for the first time that differences between sighted and blind infants in somatosensory spatial representations are located in sensory feedforward rather than post-perceptual processing stages. Specifically, Somatosensory cortical processing commences within the primary somatosensory cortex (SI) contralateral to the side of stimulation,^18^^,^^19^^,^^20^ followed by further refinement of tactile information in higher-order cortical areas. These include bilateral secondary somatosensory cortices (SII) at approximately 100 ms, the contralateral posterior parietal cortex at around 100 ms, and bilateral frontal cortices at 140–190 ms. These higher-order somatosensory areas provide online feedback to SI and other upstream components of the somatosensory processing network.^30^ The anatomical to external somatosensory remapping step in sighted infants likely reflects processing in SII, potentially driven by back-projections concerning limb posture from the posterior parietal cortex to somatosensory cortices.^22^^,^^23^^,^^24^ Indeed, this step seems to be supported by reduced activity in the posterior parietal cortex in sighted infants, whose feedback allows effective somatosensory remapping.^14^

We also investigated the processing of auditory stimuli presented on the hand both sighted and blind infants. This auditory condition partially acted as a control to check that the effects of posture on sensory processing were specific to the somatosensory modality. This was confirmed with auditory activation remaining contralateral to the presentation of the stimuli in auditory space across hand postures. In S infants, auditory ERPs are typically characterized by prominent P50 and N200 peaks, with an N100 component that typically emerges later in life, between 3 and 4 years of age. Intracerebral recordings from auditory cortical areas indicate that the temporal lobe is the main generator of the P50 component, with a contribution of the frontal lobe in the process of sensory gating.^31^ Commensurate with this, we observed a contralateral temporal-posterior positivity in response to auditory stimulation in early sensory processing, associated with behavioral responses both in SVI and S infants. This was consistent across both limb posture conditions. These results show that, in the early years of life, the earliest feedforward stages of cortical auditory processing elicit behavioral orienting responses for both SVI and S infants.

To sum up, by gathering ERP data in response to tactile stimuli, this study enables us to go beyond findings from behavioral studies to demonstrate, in sighted infants, the early development of a neural processing hierarchy in which tactile events are mapped from anatomical to external space. As predicted, in sighted infants, somatosensory ERPs showed two processing stages: an early (45–65 ms) lateralized contralateral activation followed by more bilaterally distributed components. Crucially, we found that only this later bilaterally distributed component is modulated by the posture of the infant’s limbs, and this only occurs in response to somatosensory stimuli and only in sighted infants. These findings underscore how differences in early sensory experiences lead to very different neural sensory mechanisms for representing the sensory world. Behavioral data alone are insufficient for demonstrating differences between sighted and blind infants in terms of sensory representations, as behavioral differences could be due to differences in later post-perceptual processes. Here, we showed that the differences in spatial processing between sighted and severely visually impaired infants exist at the neural level in the early stages of somatosensory processing, and that these processing differences have direct impacts on subsequent behavioral orienting responses. The demonstration of these early sensory differences, which have direct behavioral consequences, is one of the strongest indications to date of fundamental differences in the ways in which blind and sighted infants represent their spatial worlds.

### Limitations of the study

It is important for us to highlight a caveat. We cannot exclude the possibility that full visual occlusion, rather than blindness per se, might have played a role in the observed behavioral and neural differences. Although blindfolding sighted participants does not remove differences in somatosensory spatial coding between blind and sighted adults,^25^ there is the possibility that concurrent vision plays a more critical role in early life. Indeed, early in sighted development, researchers have shown that somatosensory processing is affected by the concurrent vision of the limbs.^11^ We consider this explanation unlikely, not least because the study was carried out in low luminance conditions. Irrespective, the differences we have observed between sighted and blind infants in their behavioral and neural processing, whether due to concurrent differences in visual input or differences in prior sensory experience, surely play a critical role in the subsequent perceptual development. Further research will be needed to clarify this developmental trajectory. Furthermore, it is possible that using multisensory stimuli that are congruent (i.e., provided on the same hand) could help blind individuals to develop the two-stage remapping process shown in typical individuals.^32^ This approach could open new important perspectives in the rehabilitation of known spatial impairment observed in adult blind people.^33^

## Resource availability

### Lead contact

Requests for further information and resources should be directed to and will be fulfilled by the lead contact, Helene Vitali (helene.vitali@iit.it).

### Materials availability

This study did not generate new unique materials.

### Data and code availability

The dataset reported on in this study has been deposited at Zenodo at https://zenodo.org/records/11190209 and is publicly available as of the date of publication. This article does not report original code. The processing and analysis pipeline is based on functions of tools that are publicly available (EEGLAB: https://sccn.ucsd.edu/eeglab/index.php, R: https://www.r-project.org). Any additional information required to reanalyze the data reported in this article is available from the lead contact upon request.

## Acknowledgments

The authors would like to thank Elisa Freddi and Miriana Ferro for their precious help with video scoring. This research was fully supported by the 10.13039/501100000781European Research Council (PI Monica Gori; grant agreement no. 948349).

## Author contributions

Conceptualization, M.G., H.V., C.C., and A.B.; methodology, C.C., H.V., A.B., A.T., and W.S.; investigation, A.T., M.B.A., W.S., and C.T.; writing—original draft, H.V., M.G., and C.C.; writing—review and editing, A.B. H.V., M.G., and C.C.; funding acquisition, M.G.; resources, S.S., E.C., G.G., S.S., F.T., and P.C.; supervision, M.G., C.C., A.T., M.B.A., and W.S.

All co-authors have read and approved the final version of the article.

## Declaration of interests

The authors declare no competing interests.

## STAR★Methods

### Key resources table

**Table:** 

REAGENT or RESOURCE	SOURCE	IDENTIFIER
**Deposited data**		

EEG data	Zenodo	https://zenodo.org/records/11190209
BSI clinical indices	Zenodo	https://zenodo.org/records/11190209

**Software and algorithms**		

MATLAB 2024b	Mathworks	https://www.mathworks.com/products/matlab
EEGLAB	SCCN	https://sccn.ucsd.edu/eeglab/index
Microstate EEGlab	github	https://github.com/atpoulsen/Microstate-EEGlab-toolbox
MVPALab	github	https://github.com/dlopezg/mvpalab/releases
R 4.2.3	The R Foundation for Statistical Computing Platform	https://www.r-project.org/
lme4	The Comprehensive R Archive Network (CRAN)	https://cran.r-project.org/web/packages/lme4/
Emmeans	CRAN	https://cran.r-project.org/web/packages/emmeans/

### Experimental model and study participant details

#### Participants

Twelve severely visually impaired infants (6 females, median age= 28.1 months, age range = 10-54 months) and twelve sighted infants (5 females, median age = 31 months, age range = 12-52 months) with similar ages (t(20)=0.51, p=0.61) took part in this study. The very low prevalence of severe congenital visual impairment means it is challenging to obtain large samples of visually impaired infants whilst applying our exclusion criteria (see below).

Previous studies on differing effects of posture on behavioural responses to tactile stimuli between S and SVI infants^2^ have uncovered large effects with significant differences between groups, with 10 participants in each group. Here, we were able to increase the number of participants to 12 in each group in order to improve statistical power. When using the t-test statistical test with α = 0.05 and power = 0.80, 12 participants are required to obtain a significant result if a large effect size (d = 1.2) is expected, as for the data considered in our previous study.^2^ This sample is thus appropriate to replicate previously observed behavioural effects. Because group-wise (S vs SVI) differences in ERP measures of sensory processing in infants have not been studied before, we here rely on behavioural predictions for the sample size calculation.

Informed consent was obtained from the infants’ parents before commencing the study. The testing procedure was carried out only when the infant was in a wakeful, alert, and calm state. Ethical approval was gained from the Ethics Committee of CER Liguria (268/2021 – DB id 11174) and confirmed by the Ethics Committee of Pavia Area, Fondazione IRCCS Policlinico San Matteo, Pavia (Italy). Full demographic data for all participants and clinical details of the SVI infants are provided in Table S1. Grating acuity was measured with Teller acuity cards. The Teller acuity cards are a widely-used standardized non-verbal test for measuring visual acuity in infants and young children that exploits the natural preference of infants to look toward patterns rather than a blank, homogeneous area (see^2^ for further details). None of the participants had a history of prenatal infections, fetal distress during delivery, any other perinatal distress, or any metabolic disorders. None of the participants demonstrated any cognitive impairment, and all had good general health status and were classified as normal in a clinical neurological examination. None of the participants was epileptic. All the participants demonstrated a normal psychomotor development level based on a clinical evaluation and on the Reynell-Zinkin Scale, which is specifically for visually impaired infants aged 0-4 years. Cerebral visual impairment was excluded based on anamnesis, clinical and instrumental visual function assessment, and neurological examination.

### Method details

#### Design

Four different sensory stimuli (Tactile only trials, Auditory only trials, Audiotactile congruent trials, and Audiotactile incongruent trials) with two hand postures (Uncrossed, Crossed) were presented in a completely random order. Each stimulus condition was presented on a maximum of 12 trials in total (6 in each posture), and therefore a maximum of 48 trials was presented in total across Tactile only and Auditory only conditions (24 in each posture). When possible, we repeated the whole experiment to increase the number of trials. The order of trials was kept constant across participants. In this report we present the data of the Tactile only and Auditory only conditions in both Crossed- and Uncrossed-hand postures. In the Tactile only condition, a single tactile stimulus was presented to either the infant’s right or left hand. In the Auditory only condition, a single auditory stimulus was presented to either the infant’s right or left hand. Therefore, in these conditions, the stimuli were never presented simultaneously. The Auditory only condition represents a control condition of the experimental paradigm, as the conflict effect of posture only involves tactile stimuli. Indeed, auditory stimuli are directly mapped on the external reference frame and do not undergo remapping from the body to the external environment.

#### Apparatus and materials

The apparatus and materials are replicated and described in our previous study.^2^ Here, we summarize the more important information. The child was seated on the lap of a parent, and a video camera located 80 cm in front of them recorded the participant’s movements (e.g,. hands, arms, head, and eyes). Video data were recorded at 25 frames per second for offline coding. The auditory and vibrotactile stimuli were delivered by two custom-built serial-controlled audiotactile stimulators^34^ that the experimenter placed in the infant’s palms. The vibrotactile stimulus was a continuous signal (112 Hz). The auditory stimulus was a 926 Hz pure tone pulsed at a frequency of 5 Hz. Each stimulus event lasted for 1000 ms, followed by a time period sufficient to observe the infant’s reaction to the stimulus (max ∼4000 ms). An additional visual stimulator, placed behind the infant, was recorded by the camera and provided a synchronisation signal on the video recording, allowing the offline behavioural coders to link behaviour to the stimuli presented. The signal light was illuminated when the auditory or vibrotactile stimulation began, and extinguished when the acoustic or vibrotactile stimulation ended. Importantly, this visual signal did not indicate which hand the stimulus was presented to in order to blind behavioural coders to the stimulus location.

#### EEG data acquisition

Brain electrical activity was recorded continuously by using a Hydrocel Geodesic Sensor Net, consisting of 128 silver–silver chloride electrodes evenly distributed across the scalp. The vertex served as the reference.

#### Procedure

The experiment was conducted by 2 operators, replicating our previous study,^2^ and summarized as follows. One experimenter (E1) faced the infant participant and manipulated the posture (crossed – uncrossed) of their arms. The second experimenter (E2) observed and triggered the stimuli once the infant was in the correct posture via a Matlab program, which also sent the EEG recording triggers denoting the onset/offset of specific stimulus, condition, and posture.

For each trial, E1 played a game with the infant, “bouncing” their hands into the correct position and saying “1,2,3, bù”. The hands were placed in the allocated posture just before “bù”, and the “bù” also functioned as a cue for E2 to trigger a stimulus. Following the delivery of the stimulus, E1 held the infant’s arms in the assigned posture until the infant either moved their hands. If the infant became fussy, musical toys were used to entertain them until they were calm enough to proceed with the study. The study continued for as long as the infant and parent were willing to participate, or until all trials had been completed.

### Quantification and statistical analysis

#### Observational coding of behavioural responses

The direction, latency, and type of the infants’ first orienting responses to the stimuli (auditory and tactile trials) on each trial were coded from the video records by two raters naive to the purpose of the study, as reported in our previous study.^2^ During the period following the stimulus on each trial, lateral eye movements (saccades), lateral head movements, and unilateral hand/arm movements were all coded as orienting responses. Where the infants made bilateral, symmetrical arm movements, these were coded as null responses.

The ratings provided by the two independent observers were compared and examined for agreement using Cohen’s Kappa and weighted Kappa. Unweighted and weighted Kappa agreements for response side (Left vs Right vs Null) were .942 (95% CIs: .941-.948) and .940 (95% CIs: .935-.941), respectively. Unweighted and weighted Kappa agreements for response modality (Arm/hand vs Eye/head vs Null) were .950 (95% CIs: .940-.955) and .941 (95% CIs: .929-.955), respectively. To optimize the statistical power of the data analysed, the raters first worked to reach an agreement on trials with differing ratings regarding the response side and modality. This approach enhanced inter-rater reliability. Unweighted and weighted Kappa agreements for response side (Left vs Right vs Null) were .984 (95% CIs: .974-.994) and .986 (95% CIs: .977-.995), respectively. Unweighted and weighted Kappa agreements for response modality (Arm/hand vs Eye/head vs Null) were .990 (95% CIs: .982-.998) and .993 (95% CIs: .988-.999), respectively.

#### Analyses of behavioural orienting responses

We calculated percentages of the infants’ orienting responses which were directed to the stimuli in all groups and conditions, as reported in our previous study^2^ and summarized here. Therefore, the orienting responses that we denoted as “accurate” were those where the response direction was towards (congruent with) the stimulated hand. Importantly, for the analysis, we excluded null responses (see above). For Tactile only trials, given that eye and head orienting inherently rely on an external frame of reference, we conducted additional analyses that included only hand and arm responses, excluding eye and head responses, to confirm observed effects and interactions. The direction of the infants’ responses on each trial in which they made an orienting response was recorded as a binary variable. A response directed toward the stimulated limb was coded as “1” (either a hand/arm movement using the limb where the auditory stimulus was presented or an eye/head turn toward this limb). A response directed toward the unstimulated limb was coded as “0” (either a hand/arm movement with the opposite limb or an eye/head turn toward the opposite limb). Only trials with an agreement between raters and with a defined orienting response were considered in inferential statistical analyses. All analyses were conducted using R^35^ functions. We applied a series of generalised linear mixed models (GLMMs) using a logistic link function. The model fitting was undertaken using the *glmer* function of the lme4 package.^36^ Predictors were evaluated using Type III Wald χ2 tests as implemented in the *Anova* function of the car package.^37^ We included random intercepts in all models, allowing for individual differences in baseline performance, but did not have enough power to estimate participant-specific slopes. Where multiple comparisons were used to follow up significant interactions, a Bonferroni correction was applied. Results were deemed significant when p<0.05.

Effects and interactions of Posture, Stimulus condition, Group, and Age (in months) were evaluated in the GLMMs. Significant fixed effects were further investigated with the *emmeans* function of the emmeans package^38^ by obtaining estimated marginal means (EMMs) and computing their contrasts. Effect sizes were estimated using the *eff_size* function of the emmeans package, using the sigma and the edf estimated by every single model.

The following models were fitted to the orienting responses and latency to investigate: (i) Auditory localization, (ii) Tactile localization. Note that in the analysis of tactile localization, only hand/arm responses were included. According to Wilkinson’s notation,^39^ the models fitted were:

Auditory localization (Auditory only condition): Stimulus response ∼ Group(SVI/S)∗Posture(Uncrossed/Crossed)∗Age in months+(1|participant); Tactile localization (Tactile only condition): Stimulus response ∼ Group(SVI/S)∗Posture(Uncrossed/Crossed)∗Age in months+(1|participant).

For two-way interactions, the correction was applied against all meaningful contrasts, i.e., with each level of one factor, pairwise comparisons were drawn between for each level of the other factor. So, when considering the Group x Posture interaction, for each group we compared postures (in emmeans: list(pairwise∼posture|group), n = 2 comparisons), and for each posture we compared groups (in emmeans: list(pairwise∼group|posture), n = 2 comparisons).

#### Processing and analysis of EEG data

The EEG was band-pass filtered between 0.1 and 30 Hz. Similar to recent pre-processing pipelines for infant EEG,^40^ we adopted a visually supervised automated approach in which bad channels were first removed, followed by artefact rejection tools. We identified bad channels based on the classification of flat signals or abnormal periodograms and outlier channels on each trial in each participant. Transient stereotypical (e.g., eye blinks) and non-stereotypical (e.g., movement, muscle bursts) high-amplitude artifacts were removed using an automated artifact rejection method named Artifact Subspace Reconstruction (ASR;^41^, which is available as a plug-in for EEGLAB software.^42^ ASR has also been used in other recent pre-processing pipelines for infant EEG.^40^ ASR uses a sliding window technique whereby each window of EEG data is decomposed via principal component analysis so it can be compared statistically with the whole recording considered as calibration data.^41^ Within each sliding window, the ASR algorithm identifies principal subspaces that significantly deviate from the baseline EEG and then reconstructs these subspaces using a mixing matrix computed from the baseline EEG recording. In this study, we used a sliding window of 500 ms and a threshold of 10 standard deviations to identify corrupted subspaces. Other parameters were kept at their default. EEG data were further cleaned using independent component analysis.^42^ Specifically, to select artefactual components based on quantitative criteria, we used the EEGLAB IC_Label toolbox,^43^ keeping all parameters as their default. For component rejection, the criteria reported in the corresponding validation papers were followed, mainly based on abnormal topographies and/or spectra. The data were then referenced to the average.

The EEG data were segmented across trials and participants in synchrony with the stimulus presentations to obtain ERPs, considering a period of 200 ms before the first stimulus onset as a baseline. Mean ERP amplitudes were computed by averaging the voltage in an early (45-65 ms) and in a late time window (105-120 ms).

To perform an analysis related to the physical characteristics of the stimuli, as well as to increase statistical power, separately for each group, posture, and sensory modality, we swapped the electrode montage of the conditions with stimuli provided on the left hand. Therefore, we referred to contralateral or ipsilateral response with respect to the stimulated hand.

To evaluate tactile activations, we considered centroparietal (CP) regions of interest, namely contralateral CP (channels E98, E102, E103) and ipsilateral CP (channels E41, E46, E47)^11^; while to evaluate auditory activations we considered temporal-posterior (TP) regions of interest, namely contralateral TP (channels E82, E83, E88, E89, E90, E94, E95, E96, E97, E99, E100, E101, E107, E108) and ipsilateral TP (channels E45, E50, E56, E57, E58, E63, E64, E65, E68, E69, E70, E73, E74).^44^ Effects and interactions of Posture, Laterality, Group, and Age (in months) on ERP amplitude were evaluated by applying linear mixed-model analyses (LMMs) for each time window and sensory modality. Significant fixed effects were further investigated with the *emtrends* function of the emmeans package by obtaining estimated marginal means (EMMs) and computing their contrasts. Effect sizes were estimated using the *eff_size* function of the emmeans package, using the sigma and the edf estimated by every single model. The following models were fitted to the orienting responses and latency to investigate: i) Auditory localization, ii) Tactile localization (according to Wilkinson’s notation):

Auditory localization (Auditory only condition): ERP mean ∼ Group(SVI/S)∗Posture(Uncrossed/Crossed)∗Laterality(Ipsilateral/Contralateral)+(1|participant); Tactile localization (Tactile only condition): ERP mean ∼ Group(SVI/S)∗Posture(Uncrossed/Crossed)∗Laterality(Ipsilateral/Contralateral)+(1|participant).

For two-way interactions, Bonferroni correction was applied against all meaningful contrasts, i.e., with each level of one factor, pairwise comparisons were drawn between each level of the other factor. So, when considering the Group x Laterality interaction, for each group we compared laterality (in emtrends list(pairwise∼laterality|group), n=2 comparisons), and for each laterality we compared groups (in emtrends: list(pairwise∼group|laterality), n=2 comparisons).

We applied a cluster analysis to the infants’ EEG data to identify microstates across the auditory and somatosensory ERPs (see Figure S3).^45^^,^^46^ Microstate analysis is based on empirical observations from both continuous EEG and ERPs that the electric field topography at the scalp does not vary randomly as a function of time but rather exhibits stability for tens to hundreds of milliseconds with brief intervening intervals of topographic instability. Electrical microstates in the brain are defined as successive short time periods during which the configuration of the scalp potential field remains stable, suggesting quasi-simultaneity of activity among the nodes of large-scale networks.^47^ A few prototypic microstates, which occur in a repetitive sequence across time, can be reliably identified across participants (See top Figures S3A and S3B). Therefore, it is possible to verify if the hypothesis driven definition of time windows in ERPs is supported by bottom up data concerning temporal segmentation of activity patterns in scalp topography. EEG microstates have been shown to correlate with fMRI network patterns and have been argued to provide an index of functional temporal states of the brain.^45^^,^^48^^,^^49^ Our motivation to apply microstate analysis was to validate our hypothesis regarding a two-stage anatomical-to-external somatosensory processing hierarchy. Therefore, for each group, sensory modality, and posture, we performed a microstate analysis using the Microstate EEGlab toolbox.^50^ With respect to classical ERP analysis, microstate analysis allows to better differentiate modulations in amplitude (related to overall strength of brain activity) from modulations in topography (related to specific active networks ) at a given moment.^47^^,^^51^

To assess the presence of modulations in amplitude across the whole scalp, Global Field Power (GFP) was computed, which constitutes a single, reference-independent measure of the electrical activity. GFP is the standard deviation of all electrodes at a given time. In the case of ERPs, the resultant GFP waveform is a measure of electrical activity as a function of time (See bottom Figures S3A and S3B).

Next, to assess modulations in topography, we scaled ERPs by dividing the ERP value at each time and electrode by the instantaneous GFP, thus obtaining maps that are normalized and more comparable among subjects and conditions. Then, a well-established K-means clustering method was applied to these normalized momentary topographic scalp maps. In this clustering approach, the number of random initialisations of the algorithm (restarts) was kept to 10 (default value) and we selected the best restart based on global explained variance (GEV). Temporal smoothing was applied to microstates label sequences to avoid short segments. Specifically, we imposed a minimum 10 ms duration microstate segments. The algorithm repeatedly scanned through the microstate segments and changed the label of time frames in such small segments to the next most likely microstate class, as measured by global map dissimilarity (GMD). This was done until no microstate segment was smaller than the set threshold. To decide on the number of microstates clusters we calculated, in a range between 3 and 8 microstates, four measures of fit, namely GEV, cross-validation criterion, dispersion and normalised Krzanowski-Lai criterion. We then made a qualitative decision about the optimal number of microstates based on these measures and the quality of the topographical maps of the microstates.

#### Association between ERP amplitudes and behavioural responses

The association between participants’ ERP amplitudes and behavioural orienting response performance was investigated using linear regression of individual mean ERP amplitude across trials (in each time window) against the percentage of trials in which participants oriented towards the correct hand. For each group (SVI and S), sensory modality (tactile and auditory), posture (crossed and uncrossed), laterality (Ipsilateral and Contralateral), and time windows (early and late), we performed an independent linear regression, considering ERP mean as independent and accuracy (% of correct answer) as dependent variables.

To generalize the results of this regression analysis, we also fitted a generalized linear mixed model (GLMMs) for each time window (early and late) and sensory modality (auditory and tactile). To make the data more comparable among subjects, ERP data were Z-transformed. The model fitting was undertaken using the *glmer* function of the lme4 package.^36^ Predictors were evaluated using Type III Wald χ2 tests as implemented in the *Anova* function of the car package.^37^ We included random intercepts in all models, allowing for individual differences in baseline performance, but did not have enough power to estimate participant-specific slopes. Effects and interactions of Posture, Laterality, Group, and Age (in months) were evaluated in the GLMMs. Significant fixed effects were further investigated with the *emmeans* function. Effect sizes were estimated using the *eff_size* function. According to Wilkinson’s notation,^39^ the models fitted were:

Auditory localization (Auditory only condition): Accuracy∼Group(SVI/S)∗Posture(Uncrossed/Crossed)∗Laterality(Ipsilateral/Contralateral)∗ ERP mean+(1|participant); Tactile localization (Tactile only condition): Accuracy∼Group(SVI/S)∗Posture(Uncrossed/Crossed)∗Laterality(Ipsilateral/Contralateral)∗ ERP mean+(1|participant).

For two-way interactions, Bonferroni correction was applied against all meaningful contrasts, i.e., with each level of one factor, pairwise comparisons were drawn between each level of the other factor. So, when considering the Group x Laterality interaction, for each group we compared laterality (in emmeans: list(pairwise∼laterality|group), n=2 comparisons), and for each laterality we compared groups (in emmeans: list(pairwise∼group|laterality), n=2 comparisons).

Finally, we also applied multivariate pattern analysis (MVPA) as a means to decode stimulus-related brain states in the infant participants. MVPA uses machine learning classification techniques to differentiate patterns of activation across a cluster of channels, e.g., in response to different stimuli.^29^ Here, we performed multidimensional electroencephalography analyses at the single-trial level using the MVPAlab Matlab toolbox.^52^ To improve our statistical power, for each group separately, we grouped all the trials of all the participants. Then, for each group, posture, and sensory modality, we tried to classify trials based on the accuracy of the response made, i.e., trials with correct vs incorrect responses. The classification model adopted for the decoding analysis was a Support Vector Machine (SVM) employing linear classifiers (linear kernel). Cross-validation of decoding performance against mean accuracy was based on a k-fold technique, with folds set to 5. Accuracy is a metric fast, easy to compute, and defined as the number of hits over the total number of evaluated trials. The values of feature weights, obtained after the training process of SVM models, were considered as a measure of their contribution to the model decision boundary. We applied the Haufe procedure to transform feature weights so they can be interpreted as the origin of neural processes in scalp space, which leads to more accurate predictions of orienting responses.^53^ The contribution (or importance) of each electrode to the classification performance was then evaluated at any given time point and then averaged within the time windows of interest. In order to draw statistical inferences, we applied t-tests to compare accuracy between time windows, applying Bonferroni correction for multiple comparisons.

## References

[1] Begum Ali J., Spence C., Bremner A.J. (2015). Human infants’ ability to perceive touch in external space develops postnatally. Curr. Biol..

[2] Gori M., Campus C., Signorini S., Rivara E., Bremner A.J. (2021). Multisensory spatial perception in visually impaired infants. Curr. Biol..

[3] Bremner A.J., Mareschal D., Lloyd-Fox S., Spence C. (2008). Spatial localization of touch in the first year of life: early influence of a visual spatial code and the development of remapping across changes in limb position. J. Exp. Psychol. Gen..

[4] Heed T., Azañón E. (2014). Using time to investigate space: A review of tactile temporal order judgments as a window onto spatial processing in touch. Front. Psychol..

[5] Maij F., Seegelke C., Medendorp W.P., Heed T. (2020). External location of touch is constructed post-hoc based on limb choice. eLife.

[6] Badde S., Heed T. (2023). The hands’ default location guides tactile spatial selectivity. Proc. Natl. Acad. Sci. USA.

[7] Badde S., Röder B., Heed T. (2015). Flexibly weighted integration of tactile reference frames. Neuropsychologia.

[8] Azañón E., Camacho K., Soto-faraco S. (2010). Tactile Remapping beyond Space. Eur J Neurosci.

[9] Azañón E., Soto-Faraco S. (2008). Changing Reference Frames during the Encoding of Tactile Events. Curr. Biol..

[10] Badde S., Röder B., Heed T. (2019). Feeling a Touch to the Hand on the Foot. Curr. Biol..

[11] Rigato S., Begum Ali J., Van Velzen J., Bremner A.J. (2014). The neural basis of somatosensory remapping develops in human infancy. Curr. Biol..

[12] Previc F.H. (1998). The neuropsychology of 3-D space. Psychol. Bull..

[13] Longo M.R., Azañón E., Haggard P. (2010). More than skin deep: body representation beyond primary somatosensory cortex. Neuropsychologia.

[14] Soto-Faraco S., Azañón E. (2013). Electrophysiological correlates of tactile remapping. Neuropsychologia.

[15] Heed T., Röder B. (2010). Common anatomical and external coding for hands and feet in tactile attention: evidence from event-related potentials. J. Cogn. Neurosci..

[16] Rigato S., Bremner A.J., Mason L., Pickering A., Davis R., van Velzen J. (2013). The electrophysiological time course of somatosensory spatial remapping: Vision of the hands modulates effects of posture on somatosensory evoked potentials. Eur. J. Neurosci..

[17] Eimer M., Forster B., Fieger A., Harbich S. (2004). Effects of hand posture on preparatory control processes and sensory modulations in tactile-spatial attention. Clin. Neurophysiol..

[18] Allison T., McCarthy G., Wood C.C., Darcey T.M., Spencer D.D., Williamson P.D. (1989). Human cortical potentials evoked by stimulation of the median nerve. I. Cytoarchitectonic areas generating short-latency activity. J. Neurophysiol..

[19] Allison T., McCarthy G., Wood C.C. (1992). The relationship between human long-latency somatosensory evoked potentials recorded from the cortical surface and from the scalp. Electroencephalogr. Clin. Neurophysiol..

[20] Hämäläinen H., Kekoni J., Sams M., Reinikainen K., Näätänen R. (1990). Human somatosensory evoked potentials to mechanical pulses and vibration: contributions of SI and SII somatosensory cortices to P50 and P100 components. Electroencephalogr. Clin. Neurophysiol..

[21] Mauguière F., Merlet I., Forss N., Vanni S., Jousmäki V., Adeleine P., Hari R. (1997). Activation of a distributed somatosensory cortical network in the human brain: a dipole modelling study of magnetic fields evoked by median nerve stimulation. Part II: Effects of stimulus rate, attention and stimulus detection. Electroencephalogr. Clin. Neurophysiol..

[22] Macaluso E., Frith C.D., Driver J. (2000). Modulation of Human Visual Cortex by Crossmodal Spatial Attention.

[23] Schubert R., Ritter P., Wu T., Franklin B. (2008). Spatial Attention Related SEP Amplitude Modulations Covary with BOLD Signal in S1 — A Simultaneous EEG — fMRI Study. Cereb Cortex.

[24] Buchholz V.N., Jensen O., Medendorp W.P. (2011). Multiple reference frames in cortical oscillatory activity during tactile remapping for saccades. J. Neurosci..

[25] Röder B., Rösler F., Spence C. (2004). Early Vision Impairs Tactile Perception in the Blind. Curr. Biol..

[26] Azañón E., Camacho K., Morales M., Longo M.R. (2018). The Sensitive Period for Tactile Remapping Does Not Include Early Infancy. Child Dev..

[27] Ley P., Steinberg U., Hanganu-Opatz I.L., Röder B. (2015). Event-related potential evidence for a dynamic (re-)weighting of somatotopic and external coordinates of touch during visual-tactile interactions. Eur. J. Neurosci..

[28] Azañón E., Longo M.R., Soto-Faraco S., Haggard P. (2010). The posterior parietal cortex remaps touch into external space. Curr. Biol..

[29] Haynes J.-D., Rees G. (2006). Decoding mental states from brain activity in humans. Nat. Rev. Neurosci..

[30] Uppal N., Foxe J.J., Butler J.S., Acluche F., Molholm S. (2016). The neural dynamics of somatosensory processing and adaptation across childhood : a high-density electrical mapping study. J. Neurophysiol..

[31] Korzyukov O., Pflieger M.E., Wagner M., Bowyer S.M., Rosburg T., Sundaresan K., Elger C.E., Boutros N.N. (2007). Generators of the intracranial P50 response in auditory sensory gating. Neuroimage.

[32] Gori M., Vercillo T., Sandini G., Burr D. (2014). Tactile feedback improves auditory spatial localization. Front. Psychol..

[33] Campus C., Sandini G., Amadeo M.B., Gori M. (2019). Stronger responses in the visual cortex of sighted compared to blind individuals during auditory space representation. Sci. Rep..

[34] Gori M., Amedeo M.B., Bollini A., Tonelli A., Campus C., Maviglia A., Crepaldi M., IEEE Xplore (2019). IEEE International Symposium on Medical Measurements and Applications (MeMeA).

[35] R Core Team (2024). R: A Language and Environment for Statistical Computing.

[36] Bates D., Mächler M., Bolker B., Walker S. (2015). Fitting Linear Mixed-Effects Models Using lme4. J. Stat. Softw..

[37] Fox J., Weisberg S. (2019).

[38] Lenth R.V. (2022). emmeans: Estimated Marginal Means, aka Least-Squares Means. R package. https://CRAN.R-project.org/package=emmeans.

[39] Wilkinson G.N., Rogers C.E. (1973). Symbolic Description of Factorial Models for Analysis of Variance. Appl. Stat..

[40] Kumaravel V.P., Farella E., Parise E., Buiatti M. (2022). NEAR: An artifact removal pipeline for human newborn EEG data. Dev. Cogn. Neurosci..

[41] Chang C.Y., Hsu S.H., Pion-Tonachini L., Jung T.P. (2018). Evaluation of Artifact Subspace Reconstruction for Automatic EEG Artifact Removal. Annu. Int. Conf. IEEE Eng. Med. Biol. Soc..

[42] Delorme A., Makeig S. (2004). EEGLAB: an open source toolbox for analysis of single-trial EEG dynamics including independent component analysis. J. Neurosci. Methods.

[43] Pion-Tonachini L., Kreutz-Delgado K., Makeig S. (2019). ICLabel: An automated electroencephalographic independent component classifier, dataset, and website. Neuroimage.

[44] Russo N., Foxe J.J., Brandwein A.B., Altschuler T., Gomes H., Molholm S. (2010). Multisensory processing in children with autism: High-density electrical mapping of auditory-somatosensory integration. Autism Res..

[45] Michel C.M., Koenig T. (2018). EEG microstates as a tool for studying the temporal dynamics of whole-brain neuronal networks: A review. Neuroimage.

[46] Bagdasarov A., Brunet D., Michel C.M., Gaffrey M.S. (2024). Microstate Analysis of Continuous Infant EEG: Tutorial and Reliability. Brain Topogr..

[47] Murray M.M., Brunet D., Michel C.M. (2008). Topographic ERP analyses: A step-by-step tutorial review. Brain Topogr..

[48] Habermann M., Weusmann D., Stein M., Koenig T. (2018). A student’s guide to randomization statistics for multichannel event-related potentials using Ragu. Front. Neurosci..

[49] Van De Ville D., Britz J., Michel C.M. (2010). EEG microstate sequences in healthy humans at rest reveal scale-free dynamics. Proc. Natl. Acad. Sci. USA.

[50] Poulsen A.T., Pedroni A., Langer N., Hansen L.K. (2018). Microstate EEGlab toolbox: An introductory guide. bioRxiv.

[51] McCarthy G., Wood C.C. (1985). Scalp distributions of event-related potentials: an ambiguity associated with analysis of variance models. Electroencephalogr. Clin. Neurophysiol..

[52] López-García D., Peñalver J.M.G., Górriz J.M., Ruz M. (2022). MVPAlab: A machine learning decoding toolbox for multidimensional electroencephalography data. Comput. Methods Programs Biomed..

[53] Haufe S., Meinecke F., Görgen K., Dähne S., Haynes J.-D., Blankertz B., Bießmann F. (2014). On the interpretation of weight vectors of linear models in multivariate neuroimaging. Neuroimage.

