# Supplementary material for: Vision drives the neural construction of a two-stage hierarchy of spatial processing in infancy
Source: iScience. 2025 Oct 4;28(11):113707. doi: 10.1016/j.isci.2025.113707 (PMC12589875; doi:10.1016/j.isci.2025.113707)
Supplement: Document S1. Figures S1–S4 and Table S1–S7 [file mmc1.pdf]

iScience, Volume *28*

Supplemental information

Vision drives the neural construction of a two-stage hierarchy of spatial processing in infancy

Monica Gori, Helene Vitali, Andrew J. Bremner, Alessia Tonelli, Maria Bianca Amadeo, Walter Setti, Carolina Tammurello, Sabrina Signorini, Elena Cocchi, Giuseppina Giammari, Sandra Strazzer, Francesca Tinelli, Massimiliano Serafino, Paola Camicione, and Claudio Campus

SUPPLEMENTAL INFORMATION

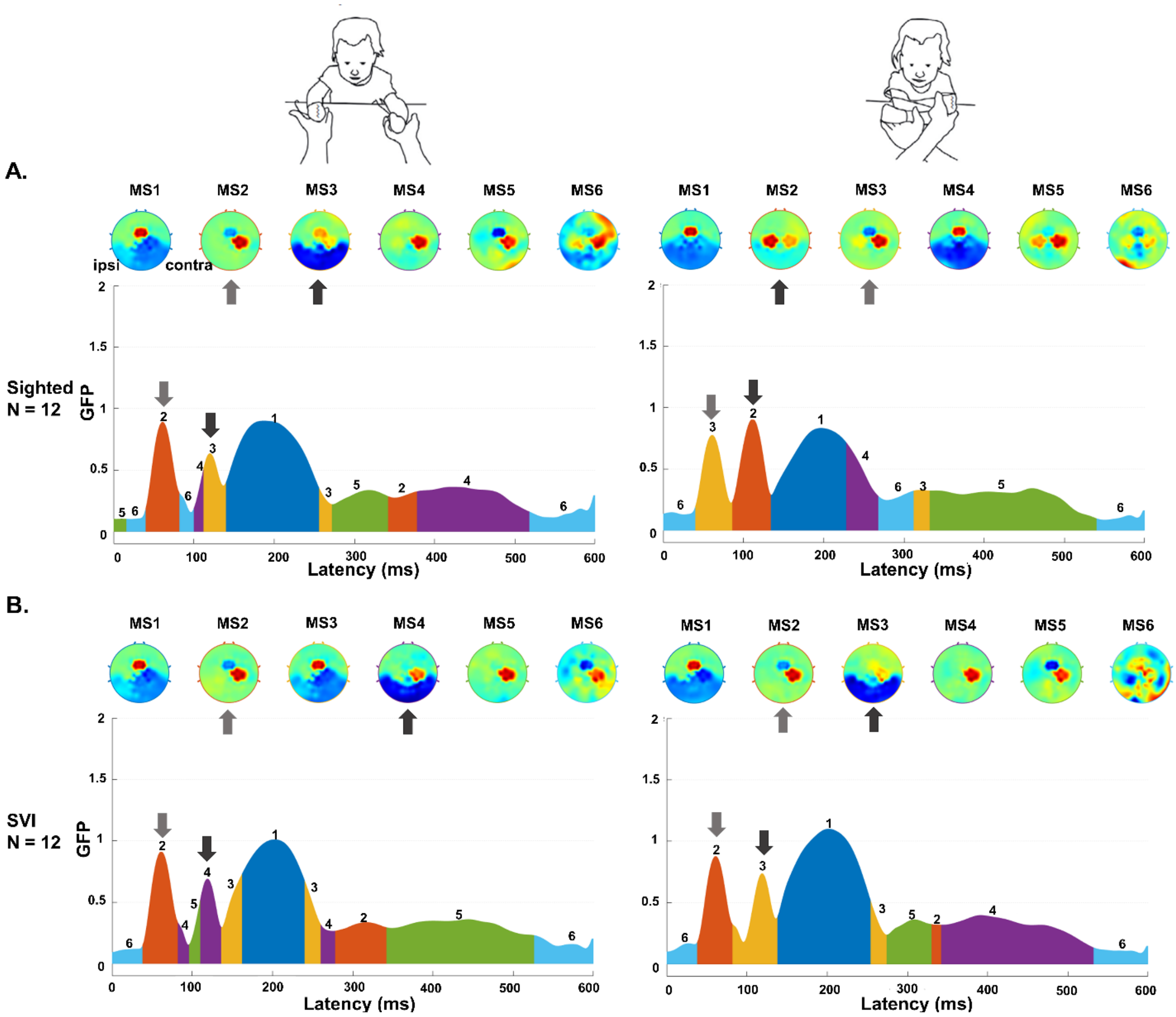

Figure S1. Microstates of tactile localization.

The results of a microstates decomposition analysis for each Group (row) and Posture (column). At the top of each subplot, we report the topographic map of each microstate. On the bottom, we report the Global Field Power (GFP) and temporal segmentation of the corresponding microstates. GFP is the standard deviation of all electrodes at a given time and represents a reference-independent measure of response strength. Microstate maps are prototypic maps obtained from ERP scalp maps scaled by GFP and clustered among subjects and represent the topography of quasi-stationary and functionally homogeneous states of the brain. Each microstate represents a time period during which the configuration of the scalp potential field remains stable, suggesting within that period a homogeneous activity of the large-scale network. A few prototypic microstates, which occur in a repetitive sequence across time, can be reliably associated with functional states. Therefore, the hypothesis-driven definition of time windows is supported by this data-driven temporal segmentation based only on the potential topography.

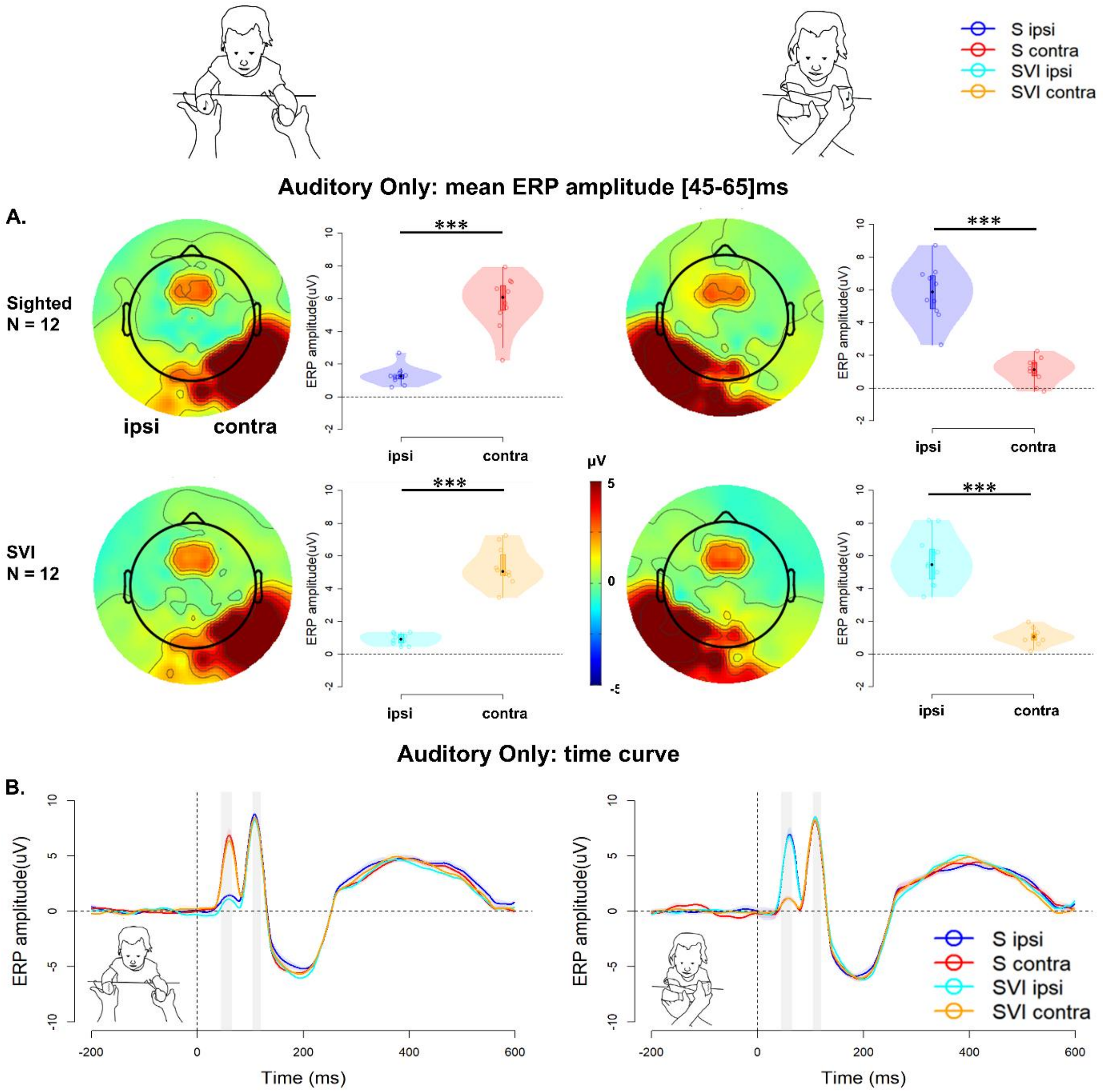

Figure S2. ERPs in the Auditory only condition across posture conditions and groups (S/SVI). (A) represents amplitude differences in the 45-65 ms time window. For each Group (row) and posture (column) we report, on the left, the topography distribution, and, on the right, the mean ERP amplitude (violin plot), the median (box plot), and related 95 % CI (vertical line), and the single-subjects ERP amplitude (scattered plot). The horizontal dashed line highlights an amplitude of 0. The asterisks represent the significant p values (*=P<0.05, **= P<0.01, ***= P<0.001) after a Bonferroni correction for multiple comparisons. (B) represents the ERP curve for different groups and hemispheres during uncrossed (left panel) and crossed (right panel) postures. The horizontal and vertical dashed lines highlight 0 amplitude and t=0, respectively. The shadowed areas highlight the time windows where amplitudes were compared.

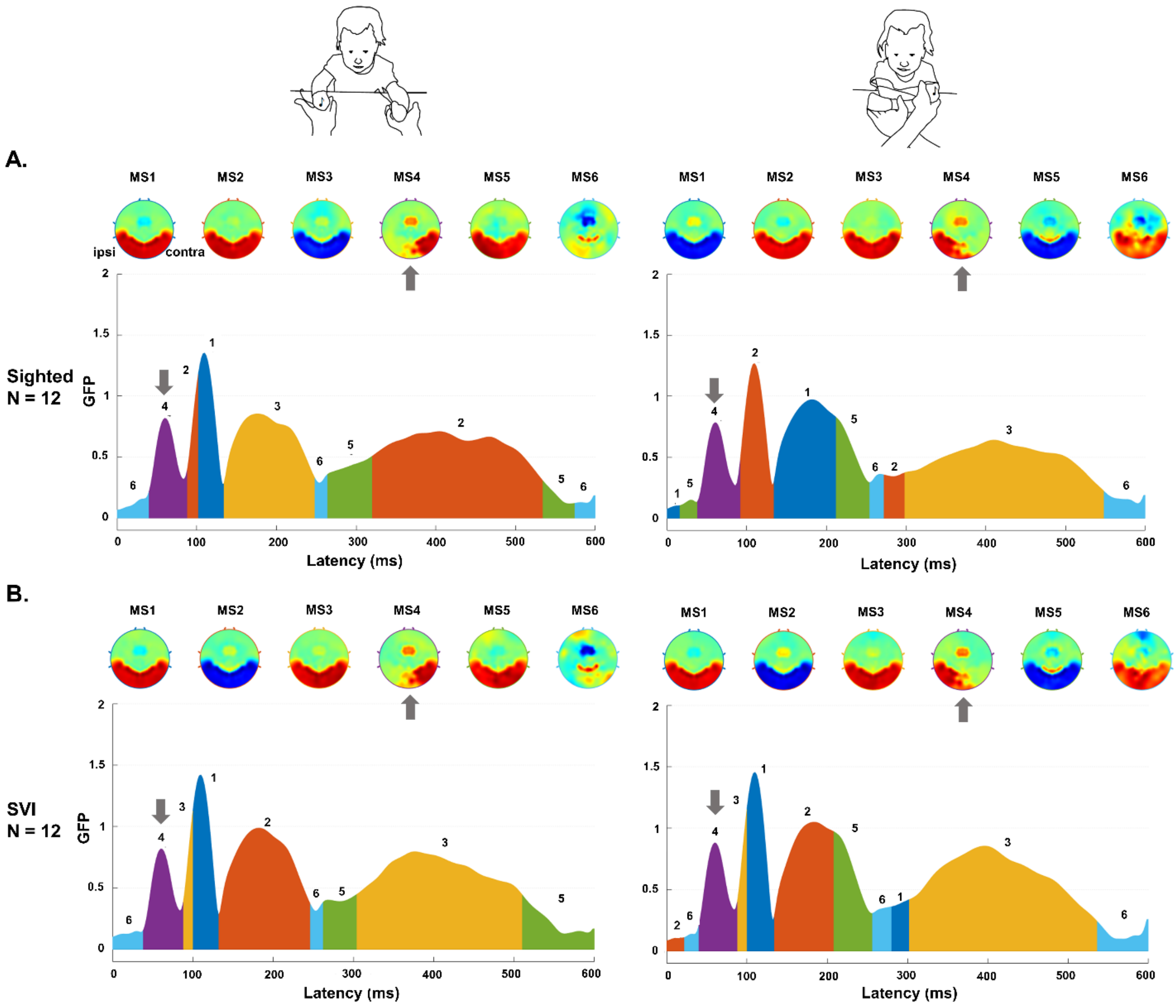

Figure S3. Microstates of auditory localization.
The results of a microstates decomposition analysis for each Group (row) and Posture (column). At the top of each subplot, we report the topographic map of each microstate. On the bottom, we report the Global Field Power (GFP) and temporal segmentation of the corresponding microstates.

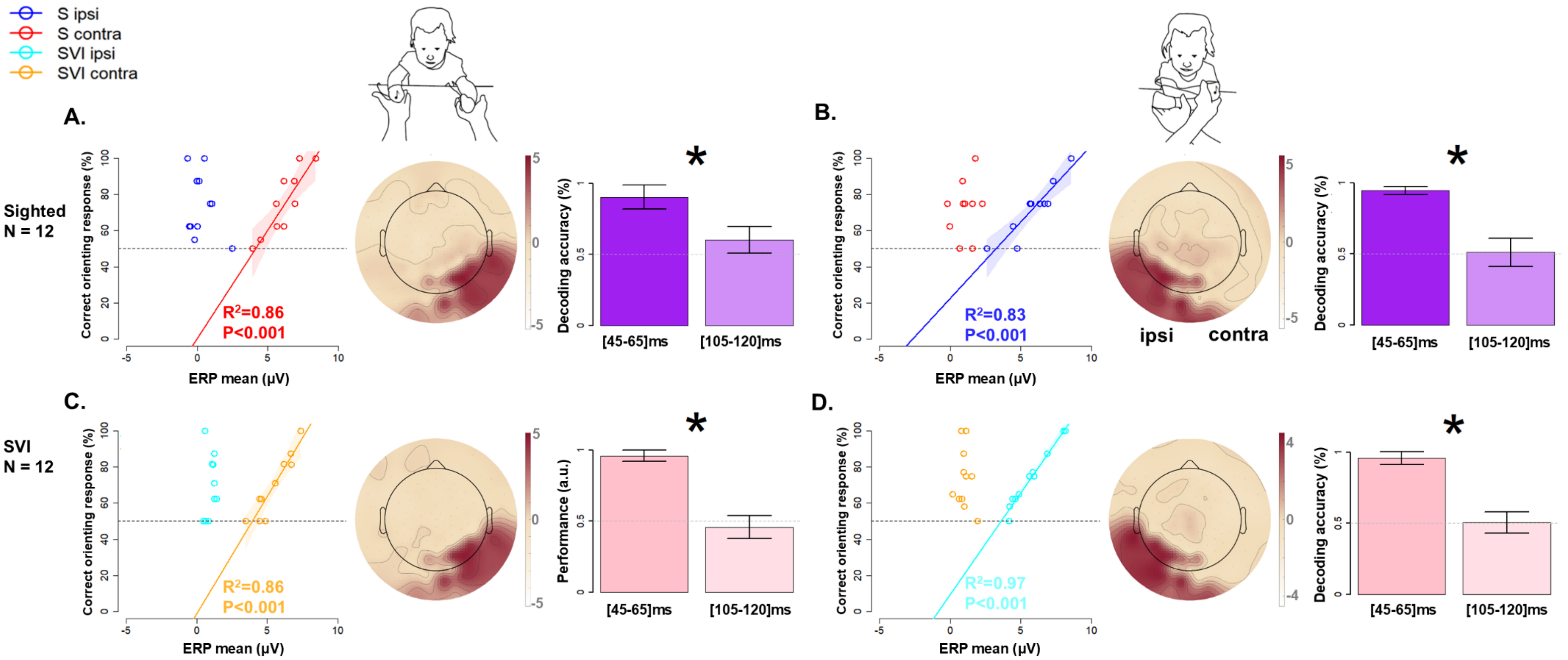

Figure S4. Auditory only condition: association between orienting accuracy and EEG in the [45-65]ms time window.
For each Group (row) and Posture (column) we report the results of linear regression of orienting accuracy to ERP amplitudes (leftmost) and MVPA classifying multivariate EEG against the accuracy of the response (middle and rightmost). The maps represent the feature weight distribution over the scalp during the selected time window, with red and white colours respectively indicating positive and negative associations with orienting performance. The rightmost subplot represents the decoding performance of the classifier (mean and SD) within the time windows. Stars indicate a significant t-test between time windows (P<0.05 after Bonferroni correction).

Table S1. Participant demographic and clinical details

Participant	Biological Sex	Age	Group	Diagnosis
001	M	10.13	Severely visually impaired	Ocular albinism
002	M	10.7	Severely visually impaired	Inherited congenital retinal dystrophy
003	F	11.53	Severely visually impaired	Eye maldevelopment
004	M	12.7	Severely visually impaired	Ocular albinism
005	M	13.07	Severely visually impaired	Inherited congenital retinal dystrophy
006	F	19.67	Severely visually impaired	Optic nerve hypoplasia
007	F	36.5	Severely visually impaired	Inherited congenital retinal dystrophy
008	M	37.23	Severely visually impaired	Inherited congenital retinal dystrophy
009	F	37.8	Severely visually impaired	Inherited congenital retinal dystrophy
010	M	38.1	Severely visually impaired	Inherited congenital retinal dystrophy
011	F	48.7	Severely visually impaired	Nystagmus
012	M	53.5	Severely visually impaired	Inherited congenital retinal dystrophy
013	M	12.17	Sighted	-
014	M	16.2	Sighted	-
015	M	21.83	Sighted	-
016	F	21.9	Sighted	-
017	F	27.5	Sighted	-
018	F	29.37	Sighted	-
019	F	32.63	Sighted	-
020	F	33	Sighted	-
021	M	35.6	Sighted	-
022	M	36.83	Sighted	-
023	M	46.43	Sighted	-
024	M	51.47	Sighted	-

This table provides details about the groups of 12 SVI and 12 S infants considered in the study. In the first column, there is the participant number; the second column has the participant's biological sex; the third column has the participant's age expressed in months; the fourth and the last columns, respectively, indicate the degree of visual impairment and the diagnosis for SVI participants.

Table S2. Percentages of participants' head and manual orienting responses

Group	Posture	Tactile only		Auditory only	
		mean	sem	mean	sem
SVI	Uncrossed	69.79	5.43	69.65	5.56
SVI	Crossed	66.36	6.23	73.90	4.97
S	Uncrossed	74.32	5.27	67.05	7.01
S	Crossed	30.45	6.05	72.50	4.86

This table provides the percentages (means and standard error of means, sem) of sighted (S) and visually impaired (SVI) infants' head and manual orienting responses, which were made toward the stimulated hand across Stimulus (tactile/auditory) and Posture (Uncrossed/Crossed) conditions.

Table S3. % Orienting response in tactile and auditory localization

Sensory Modality	***Tactile***				***Auditory***			
GLMMs	***accuracy ~ Group*Posture*Age in months + (1|participant)***				***accuracy ~ Group*Posture*Age in months + (1|participant)***			
	**χ^2(df)**		**p**		**χ^2(df)**		**p**	
Group	15.911(1)		<0.001*		0.160(1)		0.689	
Posture	21.331(1)		<0.001*		0.389(1)		0.533	
Age	0.556(1)		0.456		0.056(1)		0.813	
Group:Posture	18.899(1)		<0.001*		0.263(1)		0.608	
Group:Age	0.402(1)		0.526		0.062(1)		0.803	
Posture:Age	0.017		0.897		0.489(1)		0.484	
Group:Posture:Age	0.063		0.802		0.639(1)		0.424	
Post hoc								
Comparisons for posture	**Uncrossed**		**Crossed**		**Uncrossed**		**Crossed**	
	**t(df)**	**p**	**t(df)**	**p**	**t(df)**	**p**	**t(df)**	**p**
S-SVI contrast	0.115 (36)	0.909	-5.865 (36)	<0.001*	-	-	-	-
Comparisons for group	**S**		**SVI**		**S**		**SVI**	
	**t(df)**	**p**	**t(df)**	**p**	**t(df)**	**p**	**t(df)**	**p**
Uncrossed-crossed contrast	6.293 (19)	<0.001*	0.167 (19)	0.869	-	-	-	-

This table provides the behavioural results of the Generalized Linear Mixed Effects Models (GLMMs). Top, GLMMs applied on orienting responses. On left side, Tactile condition, on the right side Auditory condition. Top, results of Wald χ2 tests applied on GLMMs fitted to data (expressed in Wilkinson notation). Each line represents a tested effect/interaction. Bottom, results of contrasts. For each posture (Uncrossed, Crossed) t tests between groups (S, SVI); for each group, t tests between postures. In parentheses the degrees of freedom. * indicates p<0.05 after correction for multiple comparisons.

Table S4. Tactile localization ERP results

Time window	***[45-65]ms***							
	**Uncrossed**				**Crossed**			
Linear models of the ERP mean	***Erp_mean ~ laterality * group + (1|subject)***				***Erp_mean ~ laterality * group + (1|subject)***			
	**χ^2(df)**		**p**		**χ^2(df)**		**p**	
laterality	206.4(1)		<0.001*		297.6(1)		<0.001*	
Group	0.017(1)		0.898		4.991(1)		0.025*	
laterality:group	2.019(1)		0.155		11.82(1)		<0.001*	
Post hoc								
Comparisons for laterality	**Ipsi**		**Contra**		**Ipsi**		**Contra**	
	**t(df)**	**p**	**t(df)**	**p**	**t(df)**	**p**	**t(df)**	**p**
S-SVI contrast	-	-	-	-	3.868 (46)	<0.001*	-0.353 (46)	0.726
Comparisons for group	**S**		**SVI**		**S**		**SVI**	
	**t(df)**	**p**	**t(df)**	**p**	**t(df)**	**p**	**t(df)**	**p**
Ipsi-contra contrast	-10.86 (223)	<0.001*	-9.517 (223)	<0.001*	-9.214 (246)	<0.001*	-14.98 (246)	<0.001*
Time window	***[105-120]ms***							
	**Uncrossed**				**Crossed**			
Linear models of the ERP mean	***Erp_mean ~ laterality * group + (1|subject)***				***Erp_mean ~ laterality * group + (1|subject)***			
	**χ^2(df)**		**p**		**χ^2(df)**		**p**	
laterality	110.3(1)		<0.001*		2.623(1)		0.105	
group	0.417(1)		0.518		74.11(1)		<0.001*	
laterality:group	4.015(1)		0.045*		39.43(1)		<0.001*	
Post hoc								
Comparisons for laterality	**Ipsi**		**Contra**		**Ipsi**		**Contra**	
	**t(df)**	**p**	**t(df)**	**p**	**t(df)**	**p**	**t(df)**	**p**
S-SVI contrast	1.863(64)	0.067	-0.955 (64)	0.343	10.48 (65)	<0.001*	1.64 (65)	0.106
Comparisons for group	**S**		**SVI**		**S**		**SVI**	
	**t(df)**	**p**	**t(df)**	**p**	**t(df)**	**p**	**t(df)**	**p**
Ipsi-contra contrast	-5.714 (223)	<0.001*	-9.034 (223)	<0.001*	3.501 (247)	<0.001*	-5.458 (255)	<0.001*

This table provides tactile localization and remapping ERP results of the Linear Mixed Effects Models (LMMs) for each time-window and posture, which evaluate effects of laterality and group on ERP amplitude. Top, [45-65]ms time window, bottom [105-120]ms. For each time window, on the left side are reported the results of the uncrossed posture, while on the right side of the crossed posture. Top, results of Wald χ2 tests applied on LMMs fitted to data (expressed in Wilkinson notation). Each line represents a tested effect/interaction. Bottom, results of contrasts. For each hemisphere laterality (Ipsilateral, Contralateral with respect to stimulated hand) t tests between groups (S, SVI); for each group, t-tests between laterality. In parentheses are indicated the degrees of freedom. * indicate p<0.05 after correction for multiple comparisons.

Table S5. Tactile localization and remapping ERP – behavioural results

Time window	*[45-65]ms*								*[105-120]ms*							
	**Uncrossed**				**Crossed**				**Uncrossed**				**Crossed**			
Linear regression analysis																
	**S**		**SVI**		**S**		**SVI**		**S**		**SVI**		**S**		**SVI**	
	**R^2**	**p**	**R^2**	**p**	**R^2**	**p**	**R^2**	**p**	**R^2**	**p**	**R^2**	**p**	**R^2**	**p**	**R^2**	**p**
Ipsilateral - Accuracy*ERP mean	-	-	-	-	-	-	-	-	-	-	-	-	0.717	<0.001*	-	-
Contralateral - Accuracy*ERP mean	0.712	<0.001*	0.918	<0.001*	-	-	0.929	<0.001*	-	-	-	-	0.899	<0.001*	-	-
Generalized linear mixed models	*accuracy ~ laterality * group * erp_mean + (1|subject)*				*accuracy ~ laterality * group * erp_mean + (1|subject)*				*accuracy ~ laterality * group * erp_mean + (1|subject)*				*accuracy ~ laterality * group * erp_mean + (1|subject)*			
	**χ^2(df)**		**P**		**χ^2(df)**		**P**		**χ^2(df)**		**P**		**χ^2(df)**		**P**	
laterality	5.004(1)		0.025*		0.405(1)		0.525		0.001(1)		0.999		0.001(1)		0.971	
group	0.349(1)		0.554		9.455(1)		0.002*		0.362(1)		0.547		1.857(1)		0.173	
erp_mean	0.172(1)		0.679		7.897(1)		0.005*		2.572(1)		0.109		0.218(1)		0.641	
laterality*group	0.419(1)		0.517		0.491(1)		0.483		0.003(1)		0.955		0.028(1)		0.868	
laterality*erp_mean	18.02(1)		<0.001*		0.180(1)		0.671		0.69(1)		0.406		3.383(1)		0.066	
group*erp_mean	0.481(1)		0.488		1.096(1)		0.295		2.112(1)		0.146		0.202(1)		0.653	
laterality:group*erp_mean	0.143(1)		0.705		13.9 (1)		<0.001*		0.003(1)		0959		14.53(1)		<0.001*	
Post hoc GLMM																
Comparisons for laterality	**Ipsi**		**Contra**		**Ipsi**		**Contra**		**Ipsi**		**Contra**		**Ipsi**		**Contra**	
	**Z**	**p**	**Z**	**p**	**Z**	**p**	**Z**	**p**	**Z**	**p**	**Z**	**p**	**Z**	**p**	**Z**	**p**
S-SVI contrast	-	-	-	-	0.693	0.489	-3.809	<0.001*	-	-	-	-	-3.143	0.002*	2.829	0.005*
Comparisons for group	**S**		**SVI**		**S**		**SVI**		**S**		**SVI**		**S**		**SVI**	
	**Z**	**p**	**Z**	**p**	**Z**	**p**	**Z**	**p**	**Z**	**p**	**Z**	**p**	**Z**	**p**	**Z**	**p**
Ipsilateral-contralateral contrast	-	-	-	-	1.388	0.181	-3.517	<0.001*	-	-	-	-	-4.066	<0.001*	-1.279	0.2

This table provides tactile localization and remapping ERP-behavioral results of the linear regression (LM) and Generalized Linear Mixed Effects Models (GLMMs) for each time window and posture, which evaluate the association between behavioral results and ERP amplitude. Left side, [45-65]ms time window, Right side [105-120]ms. For each time window, on left Uncrossed posture, on the right Crossed posture. Top, results of LMs. Each line corresponds to the LM (expressed in Wilkinson notation), fitted to ERP data in each hemisphere laterality (Ipsilateral, Contralateral with respect to stimulated hand). Middle, results of Wald $\chi 2$ tests applied on GLMMs fitted to data (expressed in Wilkinson notation). Each line represents a tested effect/interaction. Bottom, results of contrasts. For each laterality (Ipsilateral, Contralateral with respect to stimulated hand) Z tests between groups (S, SVI); for each group, Z tests between laterality. In parentheses the degrees of freedom. * indicate $p<0.05$ after correction for multiple comparisons.

Table S6. Auditory localization results in the [45-65]ms time window

Auditory localization ERP results								
	**Uncrossed**				**Crossed**			
Linear models of the ERP mean	***Erp_mean ~ laterality * group + (1|subject)***				***Erp_mean ~ laterality * group + (1|subject)***			
	**χ^2(df)**		**p**		**χ^2(df)**		**p**	
laterality	214.3(1)		<0.001*		162.8(1)		<0.001*	
Group	0.986(1)		0.321		0.024(1)		0.878	
laterality:group	0.782(1)		0.782		0.07(1)		0.791	
Post hoc								
Comparisons for laterality	**Ipsi**		**Contra**		**Ipsi**		**Contra**	
	**t(df)**	**p**	**t(df)**	**p**	**t(df)**	**p**	**t(df)**	**p**
S-SVI contrast	-	-	-	-	-	-	-	-
Comparisons for group	**S**		**SVI**		**S**		**SVI**	
	**t(df)**	**p**	**t(df)**	**p**	**t(df)**	**p**	**t(df)**	**p**
Ipsi-contra contrast	-9.519 (274)	<0.001*	-11.12 (274)	<0.001*	8.618 (226)	<0.001*	9.411 (226)	<0.001*
Auditory localization ERP-behavioural results								
	**Uncrossed**				**Crossed**			
Linear regression analysis								
	**S**		**SVI**		**S**		**SVI**	
	**R^2**	**p**	**R^2**	**p**	**R^2**	**p**	**R^2**	**p**
Ipsi - Accuracy*ERP mean	-	-	-	-	0.828	<0.001*	0.971	<0.001*
Contra - Accuracy*ERP mean	0.858	<0.001*	0.858	<0.001*	-	-	-	-
Generalized linear mixed models	***accuracy ~ laterality * group * Erp_mean + (1|subject)***				***accuracy ~ laterality * group * Erp_mean + (1|subject)***			
	**χ^2(df)**		**P**		**χ^2(df)**		**P**	
**laterality**	1.715(1)		0.19		2.706(1)		0.1	
**group**	0.102(1)		0.749		0.296(1)		0.586	
**ERP mean**	16(1)		<0.001*		7.852(1)		0.005*	
**laterality:group**	0.032(1)		0.858		0.67(1)		0.413	
**laterality:erp_mean**	13.07(1)		<0.001*		13.8(1)		<0.001*	
**group:erp_mean**	0.134		0.716		0.146(1)		0.703	
**laterality:group:erp_mean**	0.321		0.571		0.155(1)		0.694	
Post hoc GLMM								
Comparisons for laterality	**Ipsi**		**Contra**		**Ipsi**		**Contra**	
	**Z**	**p**	**Z**	**p**	**Z**	**p**	**Z**	**p**
S-SVI contrast	0.456	0.649	-0.493	0.622	0.32	0.749	-0.445	0.656
Comparisons for group	**S**		**SVI**		**S**		**SVI**	
	**Z**	**p**	**Z**	**p**	**Z**	**p**	**Z**	**p**
Ipsi-contra contrast	-2.568	0.01*	-2.604	0.009*	2.616	0.009*	2.653	0.008*

This table provides statistics for each posture of the LMM to evaluate the effects of laterality and group on ERP amplitude (first section), and of the linear regression and GLMM to evaluate the association between behavioural results and ERP amplitude (second section). The same notations and conventions as applied as the previous tables.

Table S7. ERP responses

Group	Posture	Time window(ms)	Laterality	Tactile only		Auditory only	
				mean	sem	mean	sem
**SVI**	**Uncrossed**	**[45-65]**	ipsi	0.69	0.18	0.92	0.11
			contra	5.39	0.49	5.36	0.37
		**[105-120]**	ipsi	-0.06	0.16	7.39	0.12
			contra	2.68	0.11	7.59	0.15
	**Crossed**	**[45-65]**	ipsi	-0.31	0.44	5.72	0.46
			contra	5.27	0.49	1.06	0.14
		**[105-120]**	ipsi	-0.40	0.35	7.78	0.19
			contra	2.72	0.17	7.46	0.10
**S**	**Uncrossed**	**[45-65]**	ipsi	0.21	0.31	1.28	0.16
			contra	5.98	0.36	5.81	0.47
		**[105-120]**	ipsi	0.63	0.38	7.85	0.21
			contra	2.28	0.20	7.57	0.08
	**Crossed**	**[45-65]**	ipsi	1.28	0.11	5.82	0.54
			contra	5.41	0.20	1.09	0.25
		**[105-120]**	ipsi	6.24	0.41	7.36	0.22
			contra	3.54	0.70	7.39	0.25

This table provides the ERP mean and standard error of means (sem) for each group (SVI, S), posture (Uncrossed, Crossed), time window ([45-65]ms, [105-120]ms) and laterality (Ipsilateral, Contralateral with respect to the stimulated hand).
